# An Overview of Self-Consistent Field Calculations Within Finite Basis Sets †

**DOI:** 10.3390/molecules25051218

**Published:** 2020-03-08

**Authors:** Susi Lehtola, Frank Blockhuys, Christian Van Alsenoy

**Affiliations:** 1 Department of Chemistry, University of Helsinki, P.O. Box 55 (A. I. Virtasen aukio 1) Helsinki, Finland; 2 Department of Chemistry, University of Antwerp, Groenenborgerlaan 171, 2020 Antwerpen, Belgium; frank.blockhuys@uantwerpen.be

**Keywords:** self-consistent field theory, hartree-fock, density functional theory

## Abstract

A uniform derivation of the self-consistent field equations in a finite basis set is presented. Both restricted and unrestricted Hartree–Fock (HF) theory as well as various density functional approximations are considered. The unitary invariance of the HF and density functional models is discussed, paving the way for the use of localized molecular orbitals. The self-consistent field equations are derived in a non-orthogonal basis set, and their solution is discussed also in the presence of linear dependencies in the basis. It is argued why iterative diagonalization of the Kohn–Sham–Fock matrix leads to the minimization of the total energy. Alternative methods for the solution of the self-consistent field equations via direct minimization as well as stability analysis are briefly discussed. Explicit expressions are given for the contributions to the Kohn–Sham–Fock matrix up to meta-GGA functionals. Range-separated hybrids and non-local correlation functionals are summarily reviewed.

## 1. Introduction

Electronic structure calculations have become a cornerstone of modern-day research in chemistry and materials physics, allowing *in silico* modeling of chemical reactions and the first principles design of novel catalysts [1]. Electronic structure calculations on molecular systems most often employ the linear combination of atomic orbitals (LCAO) approach, where the molecular orbitals (MOs) are expanded in terms of atomic orbitals (AOs). Several possible alternatives for the form of the AOs are commonly used—Gaussian-type orbitals (GTOs), Slater-type orbitals (STOs), as well as numerical atomic orbitals (NAOs); see [2] for details. LCAO electronic structure calculations involve a variational minimization of the total energy with respect to the AO expansion coefficients of the MOs. Importantly, the formalism used in the LCAO approach is not restricted to AOs which are atom-centered basis functions; it can also be used, e.g., in combination with fully numerical basis functions as in the finite element approach, as has been recently demonstrated in [3,4]. Once the energy has been minimized and the corresponding wave function has been obtained, it is possible to compute a number of properties either directly from the electronic wave function (e.g., electron densities, orbital energies, molecular dipole moment), or from its response to external perturbations (nuclear magnetic shieldings, vibrational frequencies, etc.).

The mathematical foundations for spin-restricted Hartree–Fock (HF) theory within the LCAO approach were laid out independently by Roothaan and Hall [5,6]. In their seminal papers, Roothaan and Hall derived matrix equations that can be conveniently implemented on a computer as an iterative procedure. As will be seen later in Section 6, the Roothaan–Hall equations turn out to yield a generalized eigenvalue problem FC=SCE in the non-orthogonal AO basis set, which had been solved some years before by Löwdin in the context of Heitler–London theory [7].

Subsequently to the work by Roothaan and Hall, Pople and Nesbet [8] and Berthier [9] independently published the corresponding equations for an unrestricted (open-shell) HF description by an analogous scheme, without providing an explicit derivation. The Pople–Nesbet–Berthier equations assume a form similar to the Roothaan–Hall equations—constituting a coupled set of general eigenvalue equations—as will also be seen later on in the manuscript (Section 6). Restricted open-shell HF was then described by Roothaan [10]; restricted open-shell calculations will not be considered in the present work as they have been extensively reviewed by Krebs in [11] to which we refer for further details.

Density functional theory [12,13] (DFT; see also [14,15]) became popular in chemistry through the efforts of Pople and coworkers in making the method generally available to quantum chemists [16] and showing that atomization energies from DFT may agree well with experiment [17,18]. Also DFT turns out to yield self-consistent field (SCF) equations that assume the same form as in HF but with a different expression for the Fock matrix F. Pople and coworkers reported the equations necessary for solving SCF for DFT in the LCAO context up to generalized gradient approximation (GGA) functionals in [16]; an analogous derivation was also presented by Kobayashi et al in [19]. The self-consistent implementation of meta-GGA functionals was later described by Neumann, Nobes and Handy in [20]. Density functional calculations sometimes include also non-local correlation contributions; self-consistent LCAO implementations thereof have been reported by Vydrov and coworkers [21,22,23,24].

Despite the progress in and widespread success of DFT, to our knowledge, a uniform derivation of the SCF equations for HF and DFT including all the necessary expressions for the elements of the Kohn–Sham–Fock matrix up to the level of meta-GGA functionals has, up to now, not been explicitly published in the literature. This has likely contributed to the lack of complete support for meta-GGA functionals in popular quantum chemistry programs; for instance, PSI4 [25] and PySCF [26] lack support for meta-GGAs that depend on the Laplacian of the density such as the Becke–Roussel exchange functional [27], for example. This paper, therefore, presents such a derivation, yielding expressions of the DFT contributions to the Kohn–Sham–Fock matrix up to the level of meta-GGA functionals in a consistent way, facilitating the implementation of DFT in new programs.

The present derivation also has an obvious educational value. Indeed, in what follows, HF and various flavors of DFT belonging to different rungs of Jacob’s Ladder [28]—the local spin density approximation (LDA), the GGA and meta-GGA approximations—will be explicitly described in a uniform notation, making the similarities and dissimilarities between the approaches crystal clear. Facilitated by the uniform derivation, we will discuss key issues and features in the HF and DFT methodologies that arise from the mathematical formulation.

First, the basis set expansion of the molecular orbitals and the electron density is written out in Section 2. Then, the energy expression for HF and DFT is presented in Section 3, with a brief explanation of their physical content. The HF and DFT energy is shown to be invariant to rotations of the occupied and of the virtual orbitals in Section 4, allowing the construction of localized orbitals. The possibilities and drawbacks of spin-restricted calculations are discussed in Section 5. The finite-basis SCF equations are derived as generalized eigenvalue equations in Section 6. It is shown that the general eigenvalue equations can be reduced into normal eigenvalue equations by a transformation to an orthonormal basis in Section 7, and that linear dependencies in the basis can be eliminated on the way. The reason why the solution of the SCF equations amounts to a minimization of the total energy is rationalized in Section 8. Direct minimization methods are briefly introduced and stability analysis discussed in Section 9. The SCF method and direct minimization are contrasted in Section 10. Finally, the contributions to the Kohn–Sham–Fock matrix arising from various-rung DFT functionals are listed in Section 11. The article concludes with a brief summary and discussion in Section 12. Atomic units are used throughout the text.

## 2. Basis Set Expansion

In the HF and DFT approaches, the electronic wave function is written as a Slater determinant, in which the electrons occupy a set of MOs $\varphi (\vec{r})$. The MOs are expanded in terms of normalized expansion functions $\chi (\vec{r})$, which are typically AOs. Both φ and χ, as well as the LCAO coefficients $C_{\mu i}$, are typically chosen to be real in the lack of magnetic interactions that would generally make the Hamiltonian operator complex. Note, however, that the use of complex coefficients has been shown to be sometimes beneficial to describe challenging systems even in the lack of magnetic fields in Hartree–Fock (as reviewed in [29]) or DFT (as shown in [30]); complex instabilities may also arise in specialized methods beyond the SCF level, see [31] for instance. The expansion functions are generally not orthonormal

$$\int d\vec{r}\;\chi_{\mu }\left(\vec{r}\right)\chi_{\nu }\left(\vec{r}\right)=S_{\mu \nu }\neq \delta_{\mu \nu }$$

where $\delta_{\mu \nu }$ is the Kronecker delta: $\delta_{\mu \nu }=1$ if μ=ν and $\delta_{\mu \nu }=0$ otherwise. Greek letters, μ,ν,λ,σ,η, ζ and θ will be used to identify the expansion functions $\chi (\vec{r})$, whereas Roman letters, *i*, *j* and *k* will be used to identify the MOs φ. The α (spin-up) and β (spin-down) MOs are expanded separately as

(1) $$\begin{array}{c} \varphi_{i}^{\alpha }\left(\vec{r}\right)=\sum_{\mu =1}^{M}C_{\mu i}^{\alpha }\chi_{\mu }\left(\vec{r}\right) \end{array}$$

(2) $$\begin{array}{c} \varphi_{i}^{\beta }\left(\vec{r}\right)=\sum_{\mu =1}^{M}C_{\mu i}^{\beta }\chi_{\mu }\left(\vec{r}\right) \end{array}$$

Both the α and β MOs are orthonormal to themselves

$$\int d\vec{r}\;\varphi_{i}^{\alpha }\left(\vec{r}\right)\varphi_{j}^{\alpha }\left(\vec{r}\right)=\delta_{ij}\;\mathrm{and}\;\int d\vec{r}\;\varphi_{i}^{\beta }\left(\vec{r}\right)\varphi_{j}^{\beta }\left(\vec{r}\right)=\delta_{ij}$$

or equivalently within the basis set

$${\left(C^{\alpha }\right)}^{T}SC^{\alpha }=1\;\mathrm{and}\;{\left(C^{\beta }\right)}^{T}SC^{\beta }=1$$

However, the α orbitals are generally not orthonormal to the β orbitals:

$$\int d\vec{r}\;\varphi_{i}^{\alpha }\left(\vec{r}\right)\varphi_{j}^{\beta }\left(\vec{r}\right)\neq \delta_{ij}$$

or

(3) $${\left(C^{\alpha }\right)}^{T}SC^{\beta }\neq 1$$

The electron density plays a pivotal role in quantum chemistry. In line with chemistry literature, $\rho (\vec{r})$ will be used to denote the electron density at the point $\vec{r}$ in contrast to the physics notation $n(\vec{r})$ which is customary in the DFT literature. The total electron density is formed from the α and β densities, $\rho_{\alpha }$ and $\rho_{\beta }$, as $\rho \left(\vec{r}\right)=\rho_{\alpha }\left(\vec{r}\right)+\rho_{\beta }\left(\vec{r}\right)$. The spin-σ electron density can be evaluated as

(4) $$\rho_{\sigma }\left(\vec{r}\right)=\sum_{i=1}^{N_{\sigma }}{\left|\varphi_{i}^{\sigma }\left(\vec{r}\right)\right|}^{2}=\sum_{i=1}^{N_{\sigma }}\sum_{\mu \nu }C_{\mu i}^{\sigma }C_{\nu i}^{\sigma }\chi_{\mu }\left(\vec{r}\right)\chi_{\nu }\left(\vec{r}\right)=\sum_{\mu \nu }P_{\mu \nu }^{\sigma }\chi_{\mu }\left(\vec{r}\right)\chi_{\nu }\left(\vec{r}\right)$$

in which $N_{\sigma }$ is the number spin-σ electrons in the system, and the sums over basis functions $\sum_{\mu }$ and $\sum_{\nu }$ indicate $\sum_{\mu =1}^{M}$ and $\sum_{\nu =1}^{M}$, respectively; this convention for easier readability of sums over basis functions is used throughout the rest of this work.

The density matrix $P^{\sigma }$ has been defined in Equation (4) as

(5) $$P_{\mu \nu }^{\sigma }=\sum_{i=1}^{N_{\sigma }}C_{\mu i}^{\sigma }C_{\nu i}^{\sigma }$$

As is evident from the form of Equation (5), the density matrices are symmetric, $P_{\mu \nu }^{\sigma }=P_{\nu \mu }^{\sigma }$. As was already mentioned above, the total electron density is obtained from the sum of the α and β densities. Correspondingly, a total density matrix is given by

(6) $$P=P^{\alpha }+P^{\beta }$$

from which the total density can be evaluated using a relation analogous to Equation (4).

## 3. Energy Expression

The starting point for the derivation is the non-relativistic energy expression [5,8,13,16],

(7) $$E=\sum_{\mu \nu }P_{\mu \nu }H_{\mu \nu }+\frac{1}{2}\sum_{\mu \nu \lambda \sigma }P_{\mu \nu }P_{\lambda \sigma }\left(\mu \nu |\lambda \sigma \right)-\frac{a}{2}\sum_{\mu \nu \lambda \sigma }\left(P_{\mu \lambda }^{\alpha }P_{\nu \sigma }^{\alpha }+P_{\mu \lambda }^{\beta }P_{\nu \sigma }^{\beta }\right)\left(\mu \nu |\lambda \sigma \right)+b\int f\left(\vec{r}\right)d\vec{r}$$

where the electron repulsion integral (μν|λσ) is defined as

(8) $$\left(\mu \nu |\lambda \sigma \right)=\int d{\vec{r}}_{1}\int d{\vec{r}}_{2}\;\chi_{\mu }\left(\vec{r_{1}}\right)\chi_{\nu }\left(\vec{r_{1}}\right)\frac{1}{r_{12}}\;\chi_{\lambda }\left(\vec{r_{2}}\right)\chi_{\sigma }\left(\vec{r_{2}}\right)$$

and *a* and *b* are constants that define the fraction of HF exchange and the weight of the density functional approximation, respectively. The choice a=1 and b=0 corresponds to HF, whereas a=0 and b=1 yields a “pure” density functional without exact exchange such as the Perdew–Burke–Ernzerhof functional [32]. The choice a≠0 and b≠0 is the most general one, which corresponds to a hybrid functional [33] that are popular in quantum chemistry; perhaps the most famous example being the historical B3LYP functional [34].

The first term in Equation (7), which will be referred to as $E^{H}$, describes the kinetic energy of the electrons and the Coulombic attraction of the *N* nuclei in the system, with the matrix elements

(9) $$H_{\mu \nu }=\int dr\;\chi_{\mu }\left(\vec{r}\right)\left(-\frac{1}{2}\nabla^{2}+\sum_{N}\frac{Z_{N}}{r_{N}}\right)\chi_{\nu }\left(\vec{r}\right)$$

The one-electron operator in Equation (9) is commonly known as the core Hamiltonian, and the resulting $E^{H}$ is the dominating contribution to the total energy.

However, the core Hamiltonian lacks electronic interactions. These are described by the second and third terms in Equation (7), which describe the classical Coulomb and the quantum mechanical “exchange” energy, and are referred to as $E^{J}$ and $E^{K}$, respectively. The $E^{J}$ contribution to the total energy can be straightforwardly derived from the expression for the Coulomb repulsion between the electrons described by the electron density $\rho (\vec{r})$

$$E^{J}=\frac{1}{2}\int d\vec{r_{1}}\int d\vec{r_{2}}\;\rho \left(\vec{r_{1}}\right)\frac{1}{r_{12}}\rho \left(\vec{r_{2}}\right)$$

whereas the expression for the exchange energy contribution $E^{K}$ can be obtained, for instance, using Slater’s rules for a HF wave function (a=1).

The final term in Equation (7), referred to as $E^{\mathrm{XC}}$, describes the DFT exchange-correlation contributions which, alike $E^{J}$ and $E^{K}$, arise from electronic interactions. The exchange-correlation term is commonly written as

$$E^{\mathrm{XC}}=\int d\vec{r}\;f\left(\vec{r}\right)=\int d\vec{r}\;\rho \left(\vec{r}\right)\epsilon^{\mathrm{XC}}\left(\vec{r}\right)$$

where $\epsilon^{\mathrm{XC}}$ is the exchange-correlation energy density per electron. Usually $f(\vec{r})$ is a function of the electron density $\rho (\vec{r})$; it may also depend on the derivatives of $\rho (\vec{r})$ and the kinetic energy density $\tau (\vec{r})$, depending on which rung of Jacob’s Ladder [28] is used to the describe the exchange-correlation effects. The various rungs are discussed in Section 11.

## 4. Unitary Invariance

The $P^{\alpha }$ and $P^{\beta }$ matrices turn out to be invariant to rotations of the occupied orbitals among themselves. Rotating the occupied subset of the molecular orbitals φ by a orthogonal matrix U defines a new set of occupied orbitals

$$\varphi_{i}^{\alpha^{\prime }}=\sum_{k=1}^{N_{\alpha }}\varphi_{k}^{\alpha }U_{ki}$$

the MO coefficients of which can be obtained as

$$C_{\mu i}^{\alpha^{\prime }}=\sum_{k=1}^{N_{\alpha }}C_{\mu k}^{\alpha }U_{ki}$$

This can also be written in matrix notation as

(10) $$C^{\alpha^{\prime }}=C^{\alpha }U\;\mathrm{or}\;C^{\alpha^{\prime }}U^{T}=C^{\alpha }$$

The invariance to rotations in the occupied-occupied block is easy to prove, as

(11) $$P_{\mu \nu }^{\alpha^{\prime }}=\sum_{i=1}^{N_{\alpha }}C_{\mu i}^{\alpha^{\prime }}C_{\nu i}^{\alpha^{\prime }}=\sum_{ikl=1}^{N_{\alpha }}U_{ik}C_{\mu k}^{\alpha }U_{il}C_{\nu l}^{\alpha }=\sum_{kl=1}^{N_{\alpha }}\delta_{kl}C_{\mu k}^{\alpha }C_{\nu l}^{\alpha }=\sum_{k=1}^{N_{\alpha }}C_{\mu k}^{\alpha }C_{\nu k}^{\alpha }=P_{\mu \nu }^{\alpha }$$

where we have used the orthogonality of U, $U^{T}U=1=UU^{T}$.

The invariance to rotations in the occupied-occupied block can be used to fashion localized orbitals, for instance using an unitary optimization procedure [35]. Although localized orbitals are not strictly speaking observables—due to which several localization criteria have been suggested in the literature [36,37,38,39,40]—they have been shown to offer an effective way to study chemical reactions with ab initio calculations [41,42,43].

In addition to the occupied orbitals, in general there are also a number of unoccupied orbitals, which are commonly known as virtual orbitals. The number of virtual orbitals in any given calculation depends on the size of the basis set: the bigger the basis is, the more virtual orbitals there are. Because the virtual orbitals do not enter into the density matrix, the HF and DFT energy expression, Equation (7), is also invariant to rotations in the virtual-virtual block, similarly allowing their localization. However, as will be seen below in Section 9, the energy can be changed by mixing virtual orbitals into the occupied orbitals [44]. This approach provides another way to optimize the orbitals directly with, e.g., a gradient descent method, such as the geometric direct minimization method described in [45].

## 5. Spin-Restriction vs. Unrestriction

The molecular orbitals are obtained from the requirement that they minimize the total energy according to Equation (7). However, one must first choose the used spin formalism. The general choice is to use different spatial orbitals for the α and β electrons, in which case a spin-unrestricted approach is obtained. The unrestricted approach is often used even in systems in which there are an equal number of alpha and beta electrons, $N_{\alpha }=N_{\beta }$: although the spin-restricted and unrestricted descriptions often reproduce matching results for such systems near the equilibrium, only the unrestricted formalism is able to break bonds in general. The reason for this is that when molecules are stretched past the Coulson–Fischer point [46], the optimal orbitals spontaneously break spin symmetry, which can only be described in the unrestricted formalism. At variance, in the spin-restricted case the electrons occupy a common set of $N=N_{\alpha }=N_{\beta }=\left(N_{\alpha }+N_{\beta }\right)/2$ spatial orbitals. The limitation of the spatial orbitals to be the same for both spins, $C_{\mu i}=C_{\mu i}^{\alpha }=C_{\mu i}^{\beta }$, yields less variational freedom, and prevents the correct dissociation of e.g., the H${}_{2}$ molecule. As a flip side, the spin-restricted formalism affords computational savings over the unrestricted approach. The spin-restricted density matrices, Equation (5), reduce to

(12) $$P_{\mu \nu }^{\alpha }=P_{\mu \nu }^{\beta }=\frac{1}{2}P_{\mu \nu }=\sum_{i}^{N}C_{\mu i}C_{\nu i}$$

meaning, e.g., that the α and β exchange terms in Equation (7) coincide and can be simplified.

Spin-restriction is also possible in the case in which $N_{\alpha }\neq N_{\beta }$. In this case, a restricted open-shell method is obtained. Restricted open-shell methods are more involved than the spin-restricted and spin-unrestricted methods discussed in the present work. Restricted open-shell methods have been extensively discussed in [11] to which we refer for further discussion.

## 6. Self-Consistent Field Equations

Having chosen to use either spin-restricted or spin-unrestricted orbitals, one can proceed to the minimization of the energy expression in Equation (7). The energy expression depends only on the α and β density matrices $P^{\alpha }$ and $P^{\beta }$ and their sum P. The density matrices, in turn, are determined by the lowest $N_{\alpha }$ and $N_{\beta }$ molecular orbitals according to Equation (5). Because the energy expression in Equation (7) thus only depends on the density matrices $P^{\alpha }$ and $P^{\beta }$, it is expedient to use the chain rule to write, e.g.,

(13) $$\frac{\partial \;}{\partial C_{\theta k}^{\alpha }}=\sum_{\eta \zeta }\frac{\partial P_{\eta \zeta }^{\alpha }}{\partial C_{\theta k}^{\alpha }}\frac{\partial \;}{\partial P_{\eta \zeta }^{\alpha }}$$

where the partial derivative of the density matrix element $P_{\eta \zeta }^{\alpha }$ is

(14) $$\begin{array}{lll} \frac{\partial P_{\eta \zeta }^{\alpha }}{\partial C_{\theta k}^{\alpha }} & = & \frac{\partial \;}{\partial C_{\theta k}^{\alpha }}\sum_{i=1}^{N_{\alpha }}C_{\eta i}^{\alpha }C_{\zeta i}^{\alpha }\\ & = & \sum_{i=1}^{N_{\alpha }}\delta_{\theta \eta }\delta_{ki}C_{\zeta i}^{\alpha }+\sum_{i=1}^{N_{\alpha }}C_{\eta i}^{\alpha }\delta_{\theta \zeta }\delta_{ki}\\ & = & \delta_{\theta \eta }C_{\zeta k}^{\alpha }+C_{\eta k}^{\alpha }\delta_{\theta \zeta } \end{array}$$

The β orbital derivative of the total density matrix has the same form as Equation (14), where all α are replaced with β. Note that these findings hold even when the same orbitals are used for both α and β in a spin-restricted formalism, since the α and β orbitals are formally independent.

Due to the chain rule, Equation (13), all we need are the density matrix derivatives of the energy expression. We only have to calculate the derivatives of the energy expression of Equation (7) for one spin, as the energy expression is symmetric with respect to the α and β densities. It does not matter which spin we choose to be “up”; the expressions for the other spin will follow by symmetry by interchanging α↔β. The first term of Equation (7) yields simply

(15) $$\frac{\partial E^{H}}{\partial P_{\eta \zeta }^{\alpha }}=\frac{\partial }{\partial P_{\eta \zeta }^{\alpha }}\sum_{\mu \nu }P_{\mu \nu }H_{\mu \nu }=H_{\eta \zeta }$$

Next, taking the partial derivative with respect to $P_{\eta \zeta }^{\alpha }$ of the Coulomb and exchange terms in Equation (7) results in

(16) $$\begin{array}{ccc} \frac{\partial E^{J}}{\partial P_{\eta \zeta }^{\alpha }} & = & \sum_{\mu \nu }\left(P_{\mu \nu }^{\alpha }+P_{\mu \nu }^{\beta }\right)\left(\mu \nu |\eta \zeta \right)=\sum_{\mu \nu }P_{\mu \nu }\left(\mu \nu |\eta \zeta \right)=J_{\eta \zeta } \end{array}$$

where J is known as the Coulomb matrix, and

(17) $$\begin{array}{ccc} \frac{\partial E^{K}}{\partial P_{\eta \zeta }^{\alpha }} & = & -a\sum_{\mu \nu }P_{\mu \nu }^{\alpha }\left(\mu \eta |\nu \zeta \right)=-aK_{\eta \zeta }^{\alpha } \end{array}$$

where $K^{\alpha }$ is the spin-α exchange matrix, respectively. The Coulomb and exchange matrices can be used to rewrite the energy expression in Equation (7) as

(18) $$E=\sum_{\mu \nu }P_{\mu \nu }H_{\mu \nu }+\frac{1}{2}\sum_{\mu \nu }P_{\mu \nu }J_{\mu \nu }-\frac{a}{2}\sum_{\mu \nu }\left(P_{\mu \nu }^{\alpha }K_{\mu \nu }^{\alpha }+P_{\mu \nu }^{\beta }K_{\mu \nu }^{\beta }\right)+b\int f\left(\vec{r}\right)d\vec{r}$$

Note that in contrast to the Coulomb and exact exchange terms, the exchange-correlation term does not undergo simplifications, because the exchange-correlation term is not quadratic in the density matrix, as will be seen later in Section 11. For the time being, we will denote the partial derivative of $E^{\mathrm{XC}}$ with respect to $P_{\eta \zeta }^{\alpha }$ as

(19) $$\frac{\partial E^{\mathrm{XC}}}{\partial P_{\eta \zeta }^{\alpha }}=bK_{\eta \zeta }^{\mathrm{XC};\alpha }$$

as the full expressions for $K^{\mathrm{XC};\alpha }$ will be presented in Section 11. Now, collecting the partial derivatives in Equations (15)–(19) gives us the density matrix derivatives of the energy expression as

(20) $$\frac{\partial E}{\partial P_{\eta \zeta }^{\sigma }}=H_{\eta \zeta }+J_{\eta \zeta }-aK_{\eta \zeta }^{\sigma }+bK_{\eta \zeta }^{\mathrm{XC};\sigma }=F_{\eta \zeta }^{\sigma }$$

where we have identified the Kohn–Sham–Fock matrices $F^{\sigma }$, where σ denotes α or β. Because the density matrices defined by Equation (5) are symmetric, also the Fock matrices are symmetric, $F_{\eta \zeta }^{\sigma }=F_{\zeta \eta }^{\sigma }$. Note that since the Fock matrices only depend on the density matrices, also they are invariant to occupied-occupied and virtual-virtual rotations, $F^{\sigma^{\prime }}=F^{\sigma }$.

Naïvely, one would obtain the orbital derivative of the full energy expression in Equation (7) with Equations (13), (14) and (20) and then set it to zero to yield an equation for the unknown expansion coefficients $C^{\alpha }$. However, the molecular orbitals cannot be varied freely: one must make sure that the orbitals stay orthonormal during the variation, as otherwise the Pauli exclusion principle would be violated. For instance, the orthonormality condition for the α electrons is

(21) $$\int \varphi_{i}^{\alpha }\left(\vec{r}\right)\varphi_{j}^{\alpha }\left(\vec{r}\right)dr=\delta_{ij}$$

The way to enforce these conditions is to use Lagrangian multipliers $\varepsilon_{ij}$. That is, instead of the bare energy expression *E*, we will optimize the Lagrangian

(22) $$L=E-\sum_{ij}\varepsilon_{ij}^{\alpha }\left[\int d\vec{r}\varphi_{i}^{\alpha }\left(\vec{r}\right)\varphi_{j}^{\alpha }\left(\vec{r}\right)-\delta_{ij}\right]-\sum_{ij}\varepsilon_{ij}^{\beta }\left[\int d\vec{r}\varphi_{i}^{\beta }\left(\vec{r}\right)\varphi_{j}^{\beta }\left(\vec{r}\right)-\delta_{ij}\right]$$

where the sums over *i* and *j* run over all orbitals; that is, both the occupied and the virtual ones. We can see from Equation (22) that the matrices of Lagrangian multipliers $\varepsilon^{\alpha }$ and $\varepsilon^{\beta }$ can be chosen to be symmetric. For instance, if $\varepsilon^{\alpha }$ contained a symmetric part $\varepsilon_{s}^{\alpha }$ and an antisymmetric part $\varepsilon_{a}^{\alpha }$, $\varepsilon^{\alpha }=\varepsilon_{s}^{\alpha }+\varepsilon_{a}^{\alpha }$, the contribution from the antisymmetric part would vanish because it is multiplied with the orbital overlap that is symmetric.

Next, we can calculate $\partial L/\partial C_{\theta k}^{\alpha }$, where $\partial E/\partial C_{\theta k}^{\alpha }$ is given by Equations (13), (14), (20) and the derivative of the constraint term is given by

(23) $$\begin{array}{ccc} \frac{\partial }{\partial C_{\theta k}^{\alpha }}\sum_{ij}\varepsilon_{ij}^{\alpha }\left[\int d\vec{r}\varphi_{i}^{\alpha }\left(\vec{r}\right)\varphi_{j}^{\alpha }\left(\vec{r}\right)-\delta_{ij}\right] & = & \sum_{ij}\varepsilon_{ij}^{\alpha }\frac{\partial }{\partial C_{\theta k}^{\alpha }}\sum_{\eta \zeta }\left[C_{\eta i}^{\alpha }S_{\eta \zeta }C_{\zeta j}^{\alpha }-\delta_{ij}\right]\\ & = & \sum_{ij}\varepsilon_{ij}^{\alpha }\sum_{\eta \zeta }\left[\delta_{\eta \theta }\delta_{ki}C_{\zeta j}^{\alpha }+C_{\eta i}^{\alpha }\delta_{\zeta \theta }\delta_{kj}\right]S_{\eta \zeta }\\ & = & \sum_{\eta }\sum_{i}\varepsilon_{ki}^{\alpha }C_{\eta i}^{\alpha }S_{\theta \eta }+\sum_{\eta }\sum_{i}\varepsilon_{ik}^{\alpha }C_{\eta i}^{\alpha }S_{\eta \theta } \end{array}$$

where on the third line dummy summation indices have been renamed from *j* to *i* and ζ to η. The derivative can be evaluated as

(24) $$\begin{array}{ccc} \frac{\partial L}{\partial C_{\theta k}^{\alpha }} & = & \sum_{\eta \zeta }\frac{\partial E}{\partial P_{\eta \zeta }^{\alpha }}\frac{\partial P_{\eta \zeta }^{\alpha }}{\partial C_{\theta k}^{\alpha }}-\sum_{ij}\varepsilon_{ij}^{\alpha }\frac{\partial }{\partial C_{\theta k}^{\alpha }}\int d\vec{r}\varphi_{i}^{\alpha }\left(\vec{r}\right)\varphi_{j}^{\alpha }\left(\vec{r}\right)\\ & = & \sum_{\eta \zeta }F_{\eta \zeta }^{\alpha }\left[\delta_{\theta \eta }C_{\zeta k}^{\alpha }+C_{\eta k}^{\alpha }\delta_{\theta \zeta }\right]-\sum_{\zeta }\sum_{i}\varepsilon_{ki}^{\alpha }C_{\zeta i}^{\alpha }S_{\theta \zeta }-\sum_{\eta }\sum_{i}\varepsilon_{ik}^{\alpha }C_{\eta i}^{\alpha }S_{\eta \theta }\\ & = & 2\sum_{\eta }F_{\theta \eta }^{\alpha }C_{\eta k}^{\alpha }-2\sum_{\eta }\sum_{i}\varepsilon_{ki}^{\alpha }S_{\theta \eta }C_{\eta i}^{\alpha } \end{array}$$

because F and ε are symmetric, and dummy summation indices can be renamed.

The optimal orbitals satisfy the stationary condition $\partial L/\partial C_{\theta k}^{\alpha }=0$ from which

(25) $$\begin{array}{c} \sum_{\eta }F_{\theta \eta }^{\alpha }C_{\eta k}^{\alpha }=\sum_{\eta }\sum_{i}S_{\theta \eta }C_{\eta i}^{\alpha }\varepsilon_{ik}^{\alpha } \end{array}$$

Equation (25) can thus be written in matrix form as

(26) $$F^{\alpha }C^{\alpha }=SC^{\alpha }E^{\alpha }$$

where $E^{\alpha }$ is the (symmetric) matrix of Lagrangian multipliers.

Because $E^{\alpha }$ is symmetric, it can be diagonalized and it has real eigenvalues. Let us now assume that $U^{\alpha }$ is an orthogonal matrix that diagonalizes $E^{\alpha }$

(27) $$E_{ij}^{\alpha }\to E_{ij}^{\alpha^{\prime }}=\sum_{kl}U_{ki}^{\alpha }E_{kl}^{\alpha }U_{lj}^{\alpha }=\delta_{ij}\varepsilon_{i}^{\alpha }$$

where $\varepsilon^{\alpha }$ are the eigenvalues. Re-expressing the orbital coefficients $C^{\alpha }$ in terms of a new set of orbitals rotated by $U^{\alpha }$ with Equation (10), $C^{\alpha }=C^{\alpha^{\prime }}{\left(U^{\alpha }\right)}^{T}$, rewrites Equation (26) in the form

$$F^{\alpha }C^{\alpha^{\prime }}{\left(U^{\alpha }\right)}^{T}=SC^{\alpha^{\prime }}{\left(U^{\alpha }\right)}^{T}E^{\alpha }$$

that can be multiplied from the right by $U^{\alpha }$ producing

(28) $$F^{\alpha }C^{\alpha^{\prime }}=SC^{\alpha^{\prime }}E^{\alpha^{\prime }}$$

where, according to Equation (27), $E^{\alpha^{\prime }}={\left(U^{\alpha }\right)}^{T}E^{\alpha }U^{\alpha }$ is a diagonal matrix with elements $\varepsilon_{i}^{\alpha }$.

Equation (28) is almost what we want—an equation in the rotated basis that looks like Equation (26) with a diagonal $E^{\alpha^{\prime }}$—but one problem remains: the Kohn–Sham–Fock matrix is still the one corresponding to the original orbitals $C^{\alpha }$ instead of the transformed orbitals $C^{\alpha^{\prime }}$, while the orbital rotation by U that takes us from $C^{\alpha }$ to $C^{\alpha^{\prime }}$ may lead to a different Kohn–Sham–Fock matrix $F^{\alpha^{\prime }}\neq F^{\alpha }$. However, if we choose the form of U such that the occupied-virtual (ov) and virtual-occupied (vo) blocks vanish

(29) $$U=\left(\begin{array}{cc} U_{\mathrm{oo}} & U_{\mathrm{ov}}\\ U_{\mathrm{vo}} & U_{\mathrm{vv}} \end{array}\right)=\left(\begin{array}{cc} U_{\mathrm{oo}} & 0\\ 0 & U_{\mathrm{vv}} \end{array}\right)$$

then U only rotates occupied orbitals with occupied orbitals and virtual orbitals with virtual orbitals, meaning that the orbital rotation does not change the density matrix given in Equation (11). Then, the Fock matrix corresponding to the rotated orbitals coincides with that of the original orbitals, $F^{\prime }=F$, completing the proof that $E^{\alpha }$ can be chosen to be diagonal. (Occupied-virtual rotations $U_{\mathrm{ov}}$ or $U_{\mathrm{ov}}$, discussed in more detail in Section 9, are in fact here forbidden: the SCF equations were derived with the assumption that the energy is stationary, but this condition would instantly be violated by such rotations.)

We have thus obtained the Berthier–Pople–Nesbet [8,9,13,16] equations for the orbital coefficients

(30) $$\left\{\begin{array}{c} F^{\alpha }C^{\alpha }=SC^{\alpha }E^{\alpha }\\ F^{\beta }C^{\beta }=SC^{\beta }E^{\beta } \end{array}\right.$$

where the primes have become unnecessary and have been omitted for simplicity. The elements of the Kohn–Sham–Fock matrices $F^{\alpha }$ and $F^{\beta }$ are given by Equation (20), and the orbital energy matrices $E^{\alpha }$ and $E^{\beta }$ are diagonal. In the spin-restricted case [5,16] the α and β molecular orbitals coincide, leading to identical density matrices $P^{\alpha }$ and $P^{\beta }$, and identical Fock matrices $F^{\alpha }$ and $F^{\beta }$. In this case, the SCF equations simplify to the Roothaan–Hall form

(31) FC=SCE

which was already mentioned in the Introduction.

## 7. Solution of Self-Consistent Field Equations

The Roothaan–Hall and Berthier–Pople–Nesbet expressions take the form of a generalized eigenvalue equation. The conventional way to solve these equations is to re-express the (unknown) orbital coefficients in terms of a matrix X as

(32) $$C=X\tilde{C}$$

Inserting Equation (32) into the Roothaan–Hall equation, Equation (31), yields

$$FX\tilde{C}=SX\tilde{C}E$$

which can be multiplied from the left with $X^{T}$ to yield

$$X^{T}FX\tilde{C}=X^{T}SX\tilde{C}E$$

This means that the orbital transform of Equation (32) yields a new generalized eigenvalue equation

(33) $$\tilde{F}\tilde{C}=\tilde{S}\tilde{C}E$$

where $\tilde{F}=X^{T}FX$ and $\tilde{S}=X^{T}SX$. Now, if we choose X in such a way that $\tilde{S}=1$, Equation (33) reduces to a normal eigenvalue equation

(34) $$\tilde{F}\tilde{C}=\tilde{C}E$$

which can be solved with standard techniques. Then, the wanted orbital coefficients C can be calculated from $\tilde{C}$ using Equation (32).

If the basis set is well-conditioned, the matrix X can be chosen as

(35) $$X=V\Lambda^{-1/2}V^{T}$$

where V and Λ are the eigenvectors and eigenvalues of S

(36) $$S=V\Lambda V^{T}$$

This procedure is known as symmetric orthogonalization [7].

However, if a large LCAO basis is used, the atomic orbital basis functions centered on different atoms may generate linear dependencies in the basis, making the basis set expansion ambiguous. These linear dependencies can be removed with the “canonical” orthonormalization procedure [47], in which

(37) $$X=V^{\prime }{\Lambda^{\prime }}^{-1/2}$$

where only those eigenvectors $V_{i}$ with large enough eigenvalues $\lambda_{i}\geq \tau$ are included. The threshold τ is typically of the order of $\tau =10^{-7}\ldots 10^{-5}$, and its value may have a noticeable effect on, e.g., the absolute energies that result from a SCF calculation; relative energies, however, should be less sensitive to τ. If no eigenvalues fall under the threshold τ, the symmetric and canonical orthogonalization approaches become equivalent for the purposes of SCF calculations in the case of a well-conditioned basis set: both yield an orthonormal basis of the same size, which will yield the same variational ground state energy.

Unnormalized basis sets can also be handled easily by the orthogonalization procedure. Although in principle it is not necessary to normalize the individual basis functions before obtaining an orthonormal basis by Equations (35) and (37), computer linear algebra packages may fail to find the eigenvalues and eigenvectors in a reliable fashion if the basis functions have pronouncedly different norms. Moreover, missing normalization of the basis set affects the eigenvalues, which has repercussions for canonical orthogonalization. These issues can be circumvented by normalizing the overlap matrix $S\to S^{\prime }=nSn$ where $n_{ij}=S_{ii}^{-1/2}\delta_{ij}$ before using Equations (35) and (37) [3,4]. The orthogonalizer for the unnormalized basis set is obtained as X→nX; it is easy to see that this satisfies the necessary condition $X^{T}SX=1$ even though the symmetricity of X for the case of Equation (35) will be lost.

Even if S has been properly normalized, the use of the symmetric or canonical orthogonalization procedures still requires that the diagonalization of S is numerically stable. However, whenever a large number of linear dependencies exists in the basis set (e.g., a large number of diffuse functions are used or two nuclei are close together), S may become so ill-conditioned it cannot be accurately diagonalized. In such cases it is possible to reduce the size of the basis set without losing a significant amount of accuracy by an automatic procedure, see [48,49] for details.

## 8. Why Does the Self-Consistent Field Method Minimize the Energy?

The SCF equations, Equation (30) or Equation (31), offer a way to solve for the molecular orbitals described by C from a Kohn–Sham–Fock matrix F by finding its eigenvectors from Equation (34). However, the Kohn–Sham–Fock matrix depends on the density matrices, which are built from the molecular orbitals according to the Aufbau principle. In the SCF procedure, one tries to find a self-consistent solution: C yields F, whose eigenvectors are C. The procedure starts from an initial guess for the orbitals $C^{\sigma }$ or the density matrices $P^{\sigma }$, which have been recently reviewed and benchmarked in [50] to which we refer for further details.

Why does the self-consistent field procedure—diagonalizing $F^{\sigma }$ to update the orbital coefficients $C^{\sigma }$—correspond to minimization of the Hartree–Fock/Kohn–Sham energy? For simplicity, let us examine the case of HF theory. The energy expression, Equation (18), can be written in this case (a=1, b=0) as

(38) $$E=\mathrm{Tr}\;PH+\frac{1}{2}\mathrm{Tr}\;PJ-\frac{1}{2}\mathrm{Tr}\;P^{\alpha }K^{\alpha }-\frac{1}{2}\mathrm{Tr}\;P^{\beta }K^{\beta }$$

The Fock matrix elements, Equation (20), are given by

(39) $$\begin{array}{ccc} F^{\alpha } & = & H+J-K^{\alpha } \end{array}$$

(40) $$\begin{array}{ccc} F^{\beta } & = & H+J-K^{\beta } \end{array}$$

Equation (38) can be rewritten with Equations (39) and (40) as

(41) $$E=\frac{1}{2}\mathrm{Tr}\;P^{\alpha }\left(H+F^{\alpha }\right)+\frac{1}{2}\mathrm{Tr}\;P^{\beta }\left(H+F^{\beta }\right)$$

Expanding the density matrices using Equation (5) we see that Equation (41) can be written as

(42) $$E=\frac{1}{2}\sum_{i=1}^{N_{\alpha }}\left(h_{ii}^{\alpha }+f_{ii}^{\alpha }\right)+\frac{1}{2}\sum_{i=1}^{N_{\beta }}\left(h_{ii}^{\beta }+f_{ii}^{\beta }\right)$$

where the core Hamiltonian and Fock matrices have been written in the molecular orbital basis, $h^{\sigma }={\left(C^{\sigma }\right)}^{T}HC^{\sigma }$ and $f^{\sigma }={\left(C^{\sigma }\right)}^{T}F^{\sigma }C^{\sigma }$.

If one were to start the calculation from the core guess, then $\sum_{i}h_{ii}^{\alpha }$ and $\sum_{i}h_{ii}^{\beta }$ would be minimized. However, as discussed in [50], this is a horrible choice as it completely disregards electronic repulsion effects, meaning that the $\sum_{i}f_{ii}^{\alpha }$ and $\sum_{i}f_{ii}^{\beta }$ terms are far from optimal. The Roothaan step—obtaining new molecular orbitals by diagonalization of the Fock matrix—results in a minimization of the $\sum_{i}f_{ii}^{\alpha }$ and $\sum_{i}f_{ii}^{\beta }$ terms, as after diagonalization only the lowest orbitals become populated and the sum thus runs only over the lowest eigenvalues $f_{ii}^{\sigma }$. After the update, $\sum_{i}h_{ii}^{\alpha }$ and $\sum_{i}h_{ii}^{\beta }$ no longer yield their lowest possible values. However, the increase in the value of $\sum_{i}h_{ii}^{\alpha }+\sum_{i}h_{ii}^{\beta }$ should be much smaller than the decrease in the value of $\sum_{i}f_{ii}^{\alpha }+\sum_{i}f_{ii}^{\beta }$, as the Fock matrices $f^{\alpha }$ and $f^{\beta }$ also contain the core Hamiltonian. It is thus seen that Roothaan’s self-consistent field method, that is, the iterative diagonalization of the Fock matrix minimizes the energy.

However, the minimization is only valid for a fixed potential $f^{\sigma }$ in which the electrons are moving. When the orbitals are changed—as happens when f is made diagonal and its lowest eigenvectors occupied—a new Fock matrix F must be built and a new f constructed: the potential also changes with the electron density. If the orbitals were far from their optimal values, P and therefore F may change quite radically by the orbital update. This means that even though f was made diagonal in the previous iteration, it is no longer diagonally dominant after it has been updated. Indeed, the straightforward iterative diagonalization procedure often fails to converge for all but the simplest systems, because the density tends to undergo large oscillations in the naïve self-consistency cycle. To make the method usable, the convergence of the fixed-point problem of finding a C that generates F that generates C must be stabilized or accelerated in some way. This can be achieved, e.g., by damping [51,52], level shifts [53,54,55], or extrapolation [56,57,58,59]. Fractional occupations can also be used in the initial iterations to aid convergence [60].

The argument for why density functional calculations converge similarly to HF with the iterative Roothaan procedure is somewhat less obvious, because unlike HF the exchange-correlation functional is not generally quadratic in the density. However, the total energy expression *is* approximately quadratic also in DFT when one is sufficiently close to an extremal point, as is easily seen by a Taylor expansion of Equation (7). In practice the iterative procedure works well also for DFT, whose contributions to the Kohn–Sham–Fock matrix we will discuss in Section 11.

## 9. Direct Minimization of the Energy

Instead of solving the orbitals from the SCF equations, which were obtained in Section 6 from the stationary condition for the energy under the constraint of orthonormal orbitals, the orbitals can also be optimized by a direct minimization of the energy. As was discussed in Section 4, the energy expression of Equation (7) is invariant to occupied-occupied and virtual-virtual rotations. This means that if we have $o^{\sigma }$ occupied orbitals and $v^{\sigma }$ virtual orbitals from some initial guess (see possible choices in [50]) for spin σ, we can consider the energy as a function of a set of $o^{\sigma }v^{\sigma }$ rotation angles [44] by examining a rotation of the orbitals via Equation (10) by an orthogonal matrix

(43) $$U^{\sigma }\left(\theta^{\sigma }\right)=\exp\left(\begin{array}{cc} 0 & \theta^{\sigma }\\ -{\left(\theta^{\sigma }\right)}^{T} & 0 \end{array}\right)$$

where $\theta^{\sigma }$ is an $o^{\sigma }\times v^{\sigma }$ matrix containing the rotation angles. The rotation matrix determined by Equation (43) reduces to an identity matrix for vanishing rotation parameters, $\theta^{\sigma }=0$. Because the rotation matrix of Equation (43) is orthogonal, it automatically preserves the orthonormality of the orbitals, and special tricks i.e. Lagrangian multipliers are not needed to enforce this behavior.

The change in the density matrix is given by

(44) $$\frac{\partial P_{\eta \zeta }^{\sigma }}{\partial \theta_{ia}^{\sigma }}=\sum_{k=1}^{N_{\sigma }}\left[\frac{\partial C_{\eta k}^{\sigma }}{\partial \theta_{ia}^{\sigma }}C_{\zeta k}^{\sigma }+C_{\eta k}^{\sigma }\frac{\partial C_{\zeta k}^{\sigma }}{\partial \theta_{ia}^{\sigma }}\right]$$

How do the orbital coefficients change? Remembering that the first $N_{\sigma }$ orbitals are occupied, and the rest are virtual, we can write $C^{\sigma }=(\begin{array}{cc} C_{o}^{\sigma } & C_{v}^{\sigma }) \end{array}$. After an infinitesimal rotation θ≈0, the occupied orbitals change into $C_{o}^{\sigma^{\prime }}\approx C_{o}^{\sigma }-C_{v}^{\sigma }{\left(\theta^{\sigma }\right)}^{T}$, that is

(45) $$C_{\eta k}^{\sigma^{\prime }}=C_{\eta k}^{\sigma }-\sum_{b\;\mathrm{virtual}}C_{\eta b}^{\sigma }\theta_{kb}^{\sigma }$$

from which $\partial C_{\eta k}^{\sigma }/\partial \theta_{ia}^{\sigma }=-C_{\eta a}^{\sigma }\delta_{ik}$. Now the gradient of the energy with respect to rotation of the current set of orbitals can be obtained as

(46) $$\frac{\partial E}{\partial \theta_{ia}^{\sigma }}=\sum_{\eta \zeta }\frac{\partial E}{\partial P_{\eta \zeta }^{\sigma }}\frac{\partial P_{\eta \zeta }^{\sigma }}{\partial \theta_{ia}^{\sigma }}=\sum_{\eta \zeta }F_{\eta \zeta }^{\sigma }\sum_{k=1}^{N_{\sigma }}\left[\frac{\partial C_{\eta k}^{\sigma }}{\partial \theta_{ia}^{\sigma }}C_{\zeta k}^{\sigma }+C_{\eta k}^{\sigma }\frac{\partial C_{\zeta k}^{\sigma }}{\partial \theta_{ia}^{\sigma }}\right]=-\sum_{\eta \zeta }F_{\eta \zeta }^{\sigma }\left[C_{\eta a}^{\sigma }C_{\zeta i}^{\sigma }+C_{\eta i}^{\sigma }C_{\eta a}^{\sigma }\right]=-2f_{ia}^{\sigma }$$

where $f^{\sigma }={\left(C^{\sigma }\right)}^{T}F^{\sigma }C^{\sigma }$ is the Fock matrix in the MO basis. Direct minimization of Equation (7) can then be pursued using Equation (46) with, e.g., gradient descent methods. However, a proper preconditioning of the search direction is essential in order for the algorithm to be usable; see, e.g., the geometric direct minimization method described in [45]. Many other direct minimization methods for the HF or DFT energy have also been proposed, and we refer the interested reader to the vast existing literature that cannot be comprehensively cited here.

## 10. SCF vs. Direct Minimization

Having described two alternative ways for solving the orbitals, we can discuss their advantages and disadvantages. The self-consistent field method is hard to beat for systems where convergence is straightforward: a suitably stabilized and accelerated SCF procedure often converges within 10 to 20 iterations when a suitable initial guess (see [50]) has been provided. However, when the gap between the highest occupied and lowest unoccupied orbital is small, which commonly occurs in, e.g., first-row transition metal complexes, the SCF procedure may become extremely slow to converge, oscillate between two or more solutions, converge to a higher-lying solution, or even to a saddle-point solution. Namely, it is critically important to realize that even if the orbital gradient vanishes, or equivalently, that the SCF equations are fulfilled, this does not mean that the energy expression Equation (7) truly has been minimized. Because there are typically several occupied as well as virtual orbitals, the minimization problem involves a large number of degrees of freedom. In multivariate calculus, a vanishing gradient only means that the orbitals correspond to some kind of extremum of the energy: a local minimum, a saddle-point solution, or even a local maximum, although the lattermost is highly improbable in SCF calculations.

In contrast to the sometimes erratic behavior of the SCF method, direct minimization based on orbital rotations is guaranteed to converge onto an extremal point $f_{ia}=0$ per the theory of numerical analysis; this is of great worth when studying systems with complicated electronic structures for which conventional SCF algorithms fail. However, more predictable convergence does not come for free: the downside of direct minimization methods is that they carry a higher computational cost due to, e.g., the use of line searches in the orbital optimization. Direct minimization methods can also be formulated at the second order, yielding more robust convergence to a local minimum at the cost of more computational resources per iteration, see, e.g., [61,62,63]. Because direct minimization methods are based on an explicit rotation of the orbitals, they are able to always follow the same solution at variance to SCF methods where the orbital occupations are typically reset at every iteration according to the Aufbau principle. Because of this, direct minimization can lead to a solution where the Aufbau rule is violated, that is, the highest occupied orbital lies higher in energy than the lowest unoccupied orbital. Direct minimization methods can also be straightforwardly applied in more complicated electronic structure theories than self-consistent field theory. Such methods may especially include explicit dependence on the molecular orbitals, as discussed by one of the present authors in [31,64,65] for the Perdew–Zunger self-interaction correction [66] which depends explicitly on the $N_{\sigma }$ occupied orbitals, and [67] for the perfect quadruples [68] and perfect hextuples [69] models that also depend on the $N_{\sigma }$ corresponding virtual orbitals.

In order to check the character of the extremum found by the SCF procedure or a direct minimization method, it is necessary to continue the analysis to second-order changes in the energy with respect to the orbital rotations by finding the lowest eigenvalue of the Hessian matrix: if it is negative, rotating the orbitals in the direction of the corresponding eigenvector will result in a further decrease of the energy. Whenever post-HF calculations are performed, or benchmark-quality values are sought at the SCF level, stability analysis [70,71] should be used to guarantee that the wave function indeed corresponds to a local minimum. Alternatively, trust-region methods [72,73,74] can be employed to ensure that the orbitals converge onto a true local minimum.

As always in the minimization of multivariate functions, locating the global minimum is difficult, and typically the best one can hope for is to find a local minimum. Some systems permit several local electronic minima: for instance, charge transfer complexes may allow both a neutral (X ⋯ Y) as well as an ionic (X${}^{+}\cdots$ Y${}^{-}$) solution. Finding such physically motivated solutions is often straightforward by suitable manipulations of the initial guess, for instance, by constructing guesses via the superposition of atomic potentials [75,76] with the correct atomic charges. Sometimes it may also be interesting to locate saddle-point solutions, which have physical interpretations as excited states. Specific excited states can be explored within the SCF approach by replacing Aufbau population of the orbitals with overlap criteria [77,78] or with direct minimization by replacing the energy with the square of the gradient [79]; for instance, such an approach has been recently shown to predict highly accurate core spectra [80]. The full space of SCF solutions can be explored via, e.g., meta-dynamics [81].

## 11. Density Functional Contributions to Kohn–Sham–Fock Matrix

In Section 6 we derived expressions for the Kohn–Sham–Fock matrix elements for all but the density functional contribution

(47) $$E^{\mathrm{XC}}=b\int f\left(\vec{r}\right)d\vec{r}$$

which we will consider next. Hundreds of density functionals $f(\vec{r})$ of various forms have been published in the literature in the recent decades [82], and offering a comprehensive selection thereof poses a considerable challenge to quantum chemistry software developers. This problem is further exacerbated by the need to keep track with the several new functionals still being published every year. Moreover, the density functionals $f(\vec{r})$ typically carry extremely complicated functional forms, making their correct implementation painstaking work. The implementation is made even more difficult by the need to compute the first derivatives of $f(\vec{r})$ for the SCF procedure, as well as several higher-order ones for, e.g., the calculation of various properties.

Fortunately, these challenges have been obviated by freely available, portable standard implementations such as LibXC [83] and XCFun [84]. The LibXC software package strives to implement *all DFT functionals published in the literature*, and provides a uniform interface to ∼500 functionals of various forms. At present, LibXC is used by ∼30 electronic structure programs based on various numerical representations that range from basis set approaches (Gaussian-type orbitals, Slater-type orbitals, numerical atomic orbitals, finite elements, plane waves) to finite difference procedures. New functionals only have to be added once to LibXC, meaning the library is easily kept up to date, after which they become available to all programs that support the corresponding rung of functionals on Jacob’s ladder [28]. Next, we will derive the equations necessary to implement the various rungs’ functionals in the variational basis set approach.

### 11.1. LDA Functionals

The simplest density functional approximations (DFAs), belonging to the first rung of Jacob’s Ladder [28], are generally referred to as local (spin) density approximations (LDAs). These are functions of only the electron density [13]

(48) $$f\left(\vec{r}\right)=f\left(\rho_{\alpha }\left(\vec{r}\right),\rho_{\beta }\left(\vec{r}\right)\right)$$

such as the LDA exchange functional [85,86]

$$f\left(\vec{r}\right)=-\frac{3}{2}{\left(\frac{3}{4\pi }\right)}^{\frac{1}{3}}\left(\rho_{\alpha }^{4/3}\left(\vec{r}\right)+\rho_{\beta }^{4/3}\left(\vec{r}\right)\right)$$

Assuming *f* has the form of Equation (48), the resulting contribution to the Kohn–Sham–Fock matrix $F_{\mu \nu }^{\alpha ;\mathrm{XC}}=\partial E^{\mathrm{XC}}/\partial P_{\mu \nu }^{\alpha }$ can be evaluated using Equation (5) for the densities at point $\vec{r}$

(49) $$\rho_{\sigma }\left(\vec{r}\right)=\sum_{\mu \nu }P_{\mu \nu }^{\sigma }\chi_{\mu }\left(\vec{r}\right)\chi_{\nu }\left(\vec{r}\right)$$

as [16]

(50) $$F_{\mu \nu }^{\alpha ;\mathrm{LDA}}=\frac{\partial E^{\mathrm{XC}}}{\partial P_{\mu \nu }^{\alpha }}=b\int d\vec{r}\;\frac{\partial f(\vec{r})}{\partial \rho_{\alpha }}\frac{\partial \rho_{\alpha }}{\partial P_{\mu \nu }^{\alpha }}=b\int d\vec{r}\;\frac{\partial f(\vec{r})}{\partial \rho_{\alpha }}\chi_{\mu }\left(\vec{r}\right)\chi_{\nu }\left(\vec{r}\right)$$

with $F^{\beta ;\mathrm{LDA}}$ having an analogous expression. Note that if the integral is evaluated using numerical quadrature,

(51) $$F_{\mu \nu }^{\alpha ;\mathrm{LDA}}\approx b\sum_{i}w_{i}\;\frac{\partial f({\vec{r}}_{i})}{\partial \rho_{\alpha }}\chi_{\mu }\left(\vec{r_{i}}\right)\chi_{\nu }\left(\vec{r_{i}}\right)$$

Becke’s multigrid approach [87] and further developments thereof being the standard approach in LCAO programs, the expression of Equation (51) can be most efficiently formulated with matrix products. Storing the values of the basis functions at the quadrature points as a matrix

$$X_{\mu i}=\chi_{\mu }\left({\vec{r}}_{i}\right)$$

and defining a scaled version thereof as

$$Y_{\mu i}^{\alpha }=w_{i}\frac{\partial f({\vec{r}}_{i})}{\partial \rho_{\alpha }}\chi_{\mu }\left({\vec{r}}_{i}\right)$$

the Fock matrix contribution can be evaluated as simply as

$$F^{\alpha ;\mathrm{LDA}}=bX{\left(Y^{\alpha }\right)}^{T}$$

which is orders of magnitude faster than a simple *for* loop based algorithm.

### 11.2. GGA Functionals

The second rung of Jacob’s Ladder [28] is referred to as the Generalised Gradient Approximation [88] (GGA). Density functional approximations on this rung also depend on the derivatives of the density

$$f\left(\vec{r}\right)=f\left(\rho_{\alpha },\rho_{\beta },\gamma_{\alpha \alpha },\gamma_{\alpha \beta },\gamma_{\beta \beta }\right)$$

via the reduced gradients $\gamma_{\alpha \alpha }=\nabla \rho_{\alpha }\cdot \nabla \rho_{\alpha }$, $\gamma_{\alpha \beta }=\nabla \rho_{\alpha }\cdot \nabla \rho_{\beta }$, and $\gamma_{\beta \beta }=\nabla \rho_{\beta }\cdot \nabla \rho_{\beta }$, with the gradient of the density $\nabla \rho_{\sigma }$ being determined by

$$\nabla \rho_{\sigma }=\sum_{\mu \nu }P_{\mu \nu }^{\sigma }\nabla \left[\chi_{\mu }\left(\vec{r}\right)\chi_{\nu }\left(\vec{r}\right)\right]=\sum_{\mu \nu }P_{\mu \nu }^{\sigma }\left[\chi_{\nu }\left(\vec{r}\right)\nabla \chi_{\mu }\left(\vec{r}\right)+\chi_{\mu }\left(\vec{r}\right)\nabla \chi_{\nu }\left(\vec{r}\right)\right]$$

The GGA contribution to the Fock matrix is given by [16]

(52) $$\begin{array}{ccc} F_{\mu \nu }^{\alpha ;\mathrm{GGA}} & = & \frac{\partial E^{\mathrm{XC}}}{\partial P_{\mu \nu }^{\alpha }}\\ & = & \int d\vec{r}\;\left[\frac{\partial f(\vec{r})}{\partial \rho_{\alpha }}\frac{\partial \rho_{\alpha }}{\partial P_{\mu \nu }^{\alpha }}+\frac{\partial f(\vec{r})}{\partial \gamma_{\alpha \alpha }}\frac{\partial \gamma_{\alpha \alpha }}{\partial \nabla \rho_{\alpha }}\cdot \frac{\partial \nabla \rho_{\alpha }}{\partial P_{\mu \nu }^{\alpha }}+\frac{\partial f(\vec{r})}{\partial \gamma_{\alpha \beta }}\frac{\partial \gamma_{\alpha \beta }}{\partial \nabla \rho_{\alpha }}\cdot \frac{\partial \nabla \rho_{\alpha }}{\partial P_{\mu \nu }^{\alpha }}\right]=F_{\mu \nu }^{\alpha ;\mathrm{LDA}}\\ & + & \int d\vec{r}\;\left(2\frac{\partial f(\vec{r})}{\partial \gamma_{\alpha \alpha }}\nabla \rho_{\alpha }\left(\vec{r}\right)+\frac{\partial f(\vec{r})}{\partial \gamma_{\alpha \beta }}\nabla \rho_{\beta }\left(\vec{r}\right)\right)\cdot \left[\chi_{\nu }\left(\vec{r}\right)\nabla \chi_{\mu }\left(\vec{r}\right)+\chi_{\mu }\left(\vec{r}\right)\nabla \chi_{\nu }\left(\vec{r}\right)\right] \end{array}$$

The β expression can be obtained by switching α and β in Equation (52). In the restricted case, $E^{\mathrm{XC}}=\int d\vec{r}f\left(\rho ,\gamma \right)$ with γ=∇ρ·∇ρ, which leads to the DFT contributions to $F^{\mathrm{GGA}}$ given by the simpler expression

(53) $$F_{\mu \nu }^{\mathrm{GGA}}=\int d\vec{r}\;\left[\frac{\partial f(\vec{r})}{\partial \rho }\chi_{\mu }\left(\vec{r}\right)\chi_{\nu }\left(\vec{r}\right)+2\frac{\partial f(\vec{r})}{\partial \gamma }\nabla \rho \left(\vec{r}\right)\cdot \left[\chi_{\nu }\left(\vec{r}\right)\nabla \chi_{\mu }\left(\vec{r}\right)+\chi_{\mu }\left(\vec{r}\right)\nabla \chi_{\nu }\left(\vec{r}\right)\right]\right]$$

Practical implementations of Equations (52) and (53) can again be formulated using matrix products.

### 11.3. Meta-GGA Functionals

On the third rung on Jacob’s Ladder [28] are the meta-GGA (mGGA) approximations

$$f\left(\vec{r}\right)=f\left(\rho_{\alpha },\rho_{\beta },\gamma_{\alpha \alpha },\gamma_{\alpha \beta },\gamma_{\beta \beta },\tau_{\alpha },\tau_{\beta },\nabla^{2}\rho_{\alpha },\nabla^{2}\rho_{\beta }\right)$$

in which $\tau_{\sigma }$ and $\nabla^{2}\rho_{\sigma }$ are obtained as

(54) $$\begin{array}{ccc} \tau_{\sigma } & = & \frac{1}{2}\sum_{i=1}^{N_{\sigma }}{\left|\nabla \varphi_{i}\left(\vec{r}\right)\right|}^{2}=\frac{1}{2}\sum_{i=1}^{N_{\sigma }}\nabla \varphi_{i}\left(\vec{r}\right)\cdot \nabla \varphi_{i}\left(\vec{r}\right)=\frac{1}{2}\sum_{i=1}^{N_{\sigma }}\sum_{\mu \nu }C_{\mu i}^{\sigma }C_{\nu i}^{\sigma }\nabla \chi_{\mu }\left(\vec{r}\right)\cdot \nabla \chi_{\nu }\left(\vec{r}\right)\\ & = & \frac{1}{2}\sum_{\mu \nu }P_{\mu \nu }^{\sigma }\nabla \chi_{\mu }\left(\vec{r}\right)\cdot \nabla \chi_{\nu }\left(\vec{r}\right) \end{array}$$

(55) $$\begin{array}{ccc} \nabla^{2}\rho_{\sigma } & = & \sum_{\mu \nu }P_{\mu \nu }^{\sigma }\nabla^{2}\left[\chi_{\mu }\left(\vec{r}\right)\chi_{\nu }\left(\vec{r}\right)\right]\\ & = & \sum_{\mu \nu }P_{\mu \nu }^{\sigma }\left[\chi_{\mu }\left(\vec{r}\right)\nabla^{2}\chi_{\nu }\left(\vec{r}\right)+2\nabla \chi_{\mu }\left(\vec{r}\right)\cdot \nabla \chi_{\nu }\left(\vec{r}\right)+\chi_{\nu }\left(\vec{r}\right)\nabla^{2}\chi_{\mu }\left(\vec{r}\right)\right]\\ & = & \sum_{\mu \nu }P_{\mu \nu }^{\sigma }\left[\chi_{\mu }\left(\vec{r}\right)\nabla^{2}\chi_{\nu }\left(\vec{r}\right)+\chi_{\nu }\left(\vec{r}\right)\nabla^{2}\chi_{\mu }\left(\vec{r}\right)\right]+4\tau_{\sigma } \end{array}$$

The meta-GGA contributions to the Kohn–Sham–Fock matrix are straightforwardly obtained as [20]

(56) $$\begin{array}{ccc} F_{\mu \nu }^{\alpha ;\mathrm{mGGA}} & = & \frac{\partial E^{\mathrm{XC}}}{\partial P_{\mu \nu }^{\alpha }}\\ & = & F_{\mu \nu }^{\alpha ;\mathrm{GGA}}+\int d\vec{r}\;\left[\;\frac{\partial f(\vec{r})}{\partial \tau_{\alpha }}\frac{\partial \tau_{\alpha }}{\partial P_{\mu \nu }^{\alpha }}+\frac{\partial f(\vec{r})}{\partial \nabla^{2}\rho_{\alpha }\left(\vec{r}\right)}\frac{\partial \nabla^{2}\rho_{\alpha }\left(\vec{r}\right)}{\partial P_{\mu \nu }^{\alpha }}\;\right]\\ & = & F_{\mu \nu }^{\alpha ;\mathrm{GGA}}+\int d\vec{r}\;\left[\;\left(\frac{1}{2}\frac{\partial f(\vec{r})}{\partial \tau_{\alpha }}+2\frac{\partial f(\vec{r})}{\partial \nabla^{2}\rho_{\alpha }\left(\vec{r}\right)}\right)\nabla \chi_{\mu }\left(\vec{r}\right)\cdot \nabla \chi_{\nu }\left(\vec{r}\right)\right.\\ & & \;\;\;\;\;\;\;\;\;\;\;\;\;\;+\left.\frac{\partial f(\vec{r})}{\partial \nabla^{2}\rho_{\alpha }\left(\vec{r}\right)}\left[\chi_{\mu }\left(\vec{r}\right)\nabla^{2}\chi_{\nu }\left(\vec{r}\right)+\chi_{\nu }\left(\vec{r}\right)\nabla^{2}\chi_{\mu }\left(\vec{r}\right)\right]\right] \end{array}$$

which can again be expressed in terms of matrix products to achieve faster quadrature. The expressions remain formally the same in the restricted case, but the quantities correspond to the total electron density.

### 11.4. Range-Separated Hybrid Functionals

As was mentioned before, the use of non-zero values for the constants *a* and *b* in Equation (7) allows the inclusion of exact exchange effects in a DFT calculation. Such functionals represent the fourth rung of Jacob’s Ladder [28], and are generally referred to as hybrids. A further development on hybrid functionals are range-separated hybrids [89,90], in which the interelectronic interaction is divided into a short-range (sr) and a long-range (lr) part with a resolution of the identity

(57) $$\frac{1}{r_{12}}=\frac{\phi^{\mathrm{sr}}\left(r_{12}\right)}{r_{12}}+\frac{\phi^{\mathrm{lr}}\left(r_{12}\right)}{r_{12}}$$

where $\phi^{\mathrm{sr}}\left(r_{12}\right)+\phi^{\mathrm{lr}}\left(r_{12}\right)=1$. The rationale for range separation is that since density functional approximations for the exchange are based only on local information about the density, they fail to reproduce accurate estimates for, e.g., charge transfer processes. Separating the interaction by range per Equation (57) leads to a hybrid exchange functional that has four contributions

(58) $$E^{X}=a^{\mathrm{sr}}E^{\text{sr-HF}}+a^{\mathrm{lr}}E^{\text{lr-HF}}+b^{\mathrm{sr}}E^{\text{sr-DFT}}+b^{\mathrm{lr}}E^{\text{lr-DFT}}=a^{\mathrm{sr}}E^{\text{sr-HF}}+a^{\mathrm{lr}}E^{\text{lr-HF}}+E^{\mathrm{DFT}}$$

where we have stressed that since the DFT contributions are evaluated based only on the density (and possibly its derivatives), $b^{\mathrm{sr}}E^{\text{sr-DFT}}+b^{\mathrm{lr}}E^{\text{lr-DFT}}$ is nothing but a definition of a new density functional. In contrast, the HF contributions to the energy and the Kohn–Sham–Fock matrix have to be evaluated separately with range-separated ERIs

(59) $${\left(\mu \nu |\lambda \sigma \right)}^{\mathrm{sr}/\mathrm{lr}}=\int d{\vec{r}}_{1}\int d{\vec{r}}_{2}\;\chi_{\mu }\left(\vec{r_{1}}\right)\chi_{\nu }\left(\vec{r_{1}}\right)\frac{\phi^{\mathrm{sr}/\mathrm{lr}}\left(r_{12}\right)}{r_{12}}\;\chi_{\lambda }\left(\vec{r_{2}}\right)\chi_{\sigma }\left(\vec{r_{2}}\right)$$

Several kinds of range-separation kernels $\phi^{\mathrm{sr}/\mathrm{lr}}\left(r\right)$ have been proposed; however, the error function based kernel $\phi^{\mathrm{sr}}\left(r;\omega \right)=\mathrm{erfc}\;\left(\omega r\right)$, $\phi^{\mathrm{lr}}\left(r;\omega \right)=\mathrm{erf}\;\left(\omega r\right)$, where ω is the range-separation parameter, is by far the most commonly used one because it is exceedingly simple to implement in codes employing a plane-wave or Gaussian basis set [91,92]. The error function kernel is used, for instance, in the Heyd–Scuseria–Ernzerhof (HSE) functionals for solid-state calculations [91,93], as well as in the ωB97M-V [94] functional that is discussed below in Section 11.5. Some functionals based on Yukawa kernels, $\phi^{\mathrm{sr}}\left(r;\omega \right)=e^{-\lambda r}$, $\phi^{\mathrm{lr}}\left(r;\omega \right)=1-e^{-\lambda r}$, have also been published and are available in LibXC, for instance. It is important to check that the range-separation kernel used in the density functional implementation matches the one used in the computation of the range-separated ERIs in Equation (59), as the results will be incorrect otherwise.

### 11.5. Non-Local Correlation

Dispersion effects, i.e., van der Waals interactions, can be modeled in an *ab initio* DFT setting with non-local correlation functionals [95]

(60) $$E^{\mathrm{nlc}}=\int d{\vec{r}}_{1}d{\vec{r}}_{2}\rho \left({\vec{r}}_{1}\right)\Phi_{0}\left({\vec{r}}_{1},{\vec{r}}_{2}\right)\rho \left({\vec{r}}_{2}\right)$$

Because the non-local correlation energy term depends explicitly on the electron density, it also needs to be included in the SCF procedure, in principle. In contrast, empirical dispersion corrections such as Grimme’s various DFT-D approaches [96,97,98] do not depend on the electron density, and are added only as an *ad hoc* correction onto the electronic energy.

Perhaps the most accurate rung-3 and rung-4 functionals currently available [99,100,101], the pure B97M-V [102] mGGA as well as the range-separated ωB97M-V [94] hybrid mGGA, respectively, are built on top of [103] the VV10 non-local correlation functional [23] which is controlled by two adjustable parameters, *b* and *C*, which have been trained alongside the density functional in B97M-V and ωB97M-V. The results of a recent benchmark study suggest that the VV10 contributions on densities and orbital energies are negligible, and that sufficiently accurate energetics may be obtained by a one-shot evaluation of $E^{\mathrm{nlc}}$ in a post-SCF fashion [99]. Still, a rigorous minimization of the energy requires considering the effects of the non-local correlation on the wave function. Although Equation (60) does not appear to fit on the rungs of functionals discussed above, the VV10 kernel turns out to yield a GGA-type contribution to the Kohn–Sham–Fock matrix as discussed in [23], to which we refer for further details.

## 12. Summary and Discussion

We have presented an overview of self-consistent field calculations within a variational basis set formalism, and discussed the solution of the self-consistent field equations arising from Hartree–Fock as well as various levels of density functional approximations using either the traditional fixed-point equations or direct minimization, as well as various conceptual and numerical issues arising in their implementation. No assumptions have been made on the underlying basis set in the present work: the self-consistent field formalism is the same regardless of the form of the basis functions, which can be chosen to be, e.g., Gaussian-type orbitals (GTOs), Slater-type orbitals (STOs), numerical atomic orbitals (NAOs), or finite element shape functions. The basis set is only reflected in the molecular integrals, that is, the matrix elements of the core Hamiltonian and the two-electron integrals. The various choices for the basis set have different advantages and disadvantages, including the evaluation of the molecular integrals; see [2] for discussion. Instead, the main restriction in the present work is the implicit assumption that the basis set is compact enough so that the N×N density and Fock matrices are small enough to allow $O(N^{2})$ dense matrix storage and $O(N^{3})$ diagonalization. Although the present overview has focused on non-relativistic calculations on molecules, the discussion for relativistic calculations as well as crystalline systems is analogous to a large degree. We hope that the present, consistent and thorough derivation and analysis will be useful for reference as well as teaching purposes, and that the results presented herein will lead to a wider availability of density functionals in electronic structure programs.
